# Cross protection to SARS-CoV-2 variants in hamsters with naturally-acquired immunity

**DOI:** 10.1186/s12985-023-02136-6

**Published:** 2023-07-28

**Authors:** Saina Beitari, Diana Duque, Jegarubee Bavananthasivam, Melissa Hewitt, Jagdeep K. Sandhu, Ita Hadžisejdić, Anh Tran

**Affiliations:** 1 Infectious Diseases, Human Health Therapeutics Research Centre, National Research Council Canada, ON Ottawa, Canada; 2 Preclinical Imaging, Human Health Therapeutics Research Centre, National Research Council Canada, ON Ottawa, Canada; 3 Clinical Department of Pathology and Cytology, University of Rijeka, Rijeka, Croatia

**Keywords:** SARS-CoV-2, Variants of concern, Hamster, Cross-immunity, Original antigenic sin

## Abstract

Since SARS-CoV-2 was first reported in late 2019, multiple variations of the original virus have emerged. Each variant harbors accumulations of mutations, particularly within the spike glycoprotein, that are associated with increased viral transmissibility and escape immunity. The different mutations in the spike protein of different variants shape the subsequent antibody and T cell responses, such that exposure to different spike proteins can result in reduced or enhanced responses to heterologous variants further down the line. Globally, people have been exposed and re-exposed to multiple variations of the Ancestral strain, including the five variants of concerns. Studies have shown that the protective immune response of an individual is influenced by which strain or combination of strains they are exposed to. The initial exposure to a specific strain may also shape their subsequent immune patterns and response to later infections with a heterologous virus. Most immunological observations were carried out early during the pandemic when the Ancestral strain was circulating. However, SARS-CoV-2 variants exhibit varying patterns of disease severity, waning immunity, immune evasion and sensitivity to therapeutics. Here we investigated the cross-protection in hamsters previously infected with a variant of concern (VOC) and subsequently re-infected with a heterologous variant. We also determined if cross-protection and immunity were dependent on the specific virus to which the hamster was first exposed. We further profiled the host cytokine response induced by each SARS-CoV-2 variants as well as subsequent to re-infection. A comparative analysis of the three VOCs revealed that Alpha variant was the most pathogenic VOC to emerge. We showed that naturally acquired immunity protected hamsters from subsequent re-infection with heterologous SARS-CoV-2 variant, regardless which variant the animal was first exposed to. Our study supports observations that heterologous infection of different SARS-CoV-2 variants do not exacerbate disease in subsequent re-infections. The continual emergence of new SARS-CoV-2 variants mandates a better understanding of cross-protection and immune imprinting in infected individuals. Such information is essential to guide vaccine strategy and public policy to emerging SARS-CoV-2 VOCs and future novel pandemic coronaviruses.

## Introduction

Since the World Health Organization’s declaration of COVID-19 as a pandemic in March 2020 [13], the world has seen the emergence of a multiple variations of the original virus, five of which have been declared as variants of concerns (VOCs) and many more considered to be variants of interest. These SARS-CoV-2 variants harbor accumulations of mutations, predominantly within the spike glycoprotein, which are associated with increased viral transmissibility and escape immunity [9]. The ability for these mutations to permit escape from host immunity raised the question of immune memory durability, which could lead to reinfections and break through infections [17, 38]. While it is known that antibody levels decline over time following vaccination or infection [21, 30], an individual’s protective immune responses are also influenced by the strain or combination of strains to which they have been exposed [42, 58]. In certain cases, cross-reactive immune responses from re-infections may positively impact disease outcomes by promoting beneficial cross-reactive T cell responses [34, 40], but, in other cases, such as dengue virus and zika virus infections disease severity is potentially exacerbated [15, 61]. Now more than 3-years into the pandemic, people across the world have very different patterns of immunity to the SARS-CoV-2 virus, based on their exposure and vaccination. Globally, people have been exposed to the original strain (hereon referred to as Ancestral) and/or Alpha, Beta, Gamma, Delta and now Omicron (and all the Omicron subvariants). People may be unvaccinated or have had one or more vaccine doses [4]. The particular virus strain they are first exposed, whether by natural infection or vaccination, may also shape their subsequent immune patterns, affecting their susceptibility to infection to heterologous strains and disease severity [2, 31, 35]. The different mutations in the spike protein shape the subsequent antibody and T cell responses [25], which can result in reduced or enhanced responses to variants further down the line. This has important implications for future proofing vaccine design and dosing strategies.

Although SARS-CoV-2 is considered as pathogenic [18], COVID-19 shows a diverse range of symptoms from majority of patients reported as asymptomatic or mild disease, to severe cases that include acute respiratory distress syndrome, pneumonia, cardiac arrythmia, encephalopathy, and death [55]. COVID-19 severity and disease outcomes have been associated with various immunological correlates [5, 57] that include autoantibodies to type I interferons [52]; altered myeloid cell populations such as elevated numbers of immature neutrophils and loss of non-classical monocytes [45, 60]. Increased hyperinflammatory responses and aberrant CD163 + monocytes have also been reported [19]. Elevated serum cytokine levels are also a strong predictor of severe COVID-19 and adverse disease outcomes [16]. Similarly, certain T cell responses including unconventional CD16 + T cells, mucosal-associated invariant T (MAIT) cells and γδ T cell may contribute to immunopathology observed in increased COVID-19 disease severity [36]. Conversely, the development of a coordinated SARS-CoV-2-specific CD4 + and CD8 + T cell response and neutralizing antibodies are associated with reduced COVID-19 disease severity [44, 46]. Most immunological observations were carried out early during the pandemic when the Ancestral strain was circulating. However, SARS-CoV-2 variance have exhibited variation in disease severity, patterns and waning of immunity, immune evasion and sensitivity to therapeutics [9, 26].

Our study was initiated during the period of the pandemic when only three VOCs first emerged, so the investigation was limited to a focus on variants Alpha, Beta, and Gamma. We investigated the cross-protection in hamsters previously infected with a VOC and subsequently re-infected with a heterologous variant. We also determined if cross-protection and immunity was dependent on the specific virus to which the hamster was first exposed. We further profiled the host cytokine response induced by each SARS-CoV-2 variant as well as subsequent to re-infection. A comparative analysis of the three VOCs revealed that Alpha variant was the most pathogenic VOC to emerge. We showed that naturally acquired immunity protected hamsters from subsequent re-infection with heterologous SARS-CoV-2 variant, regardless which variant the animal was first exposed to. Our study supports observations that heterologous infection of different SARS-CoV-2 variants do not exacerbate disease in subsequent re-infections. The continual emergence of new SARS-CoV-2 variance mandates a better understanding of cross-protection and immune imprinting in infected individuals. This will contribute to our understanding of the heterogeneity of clinical outcomes in COVID-19 disease and allows us to identify conserved immune epitopes. Such information is essential to guide vaccine strategy and public policy to emerging SARS-CoV-2 VOCs and future novel pandemic coronaviruses.

## Materials and methods

### Animals and viruses

Golden Syrian hamsters, 7–8 weeks old (81–90 g) males/females were purchased from the Charles River Laboratories (Saint-Constant, Canada). Animals were maintained at the small animal facility of the National Research Council Canada (NRC) in accordance with the guidelines of the Canadian Council on Animal Care. All procedures performed on animals in this study were in accordance with regulations and guidelines reviewed and approved in animal use protocol 2020.06 by the NRC Human Health Therapeutics Animal Care Committee. Hamsters were anesthetized by injection of Ketamine/Xylazine (90 kg/mg/8 kg/mg) and intranasally challenged with 8.5 × 10^4^ plaque forming unit (PFU) of SARS-CoV-2 (100 µl per animal) or 100 µl of sterile phosphate buffered saline (1X PBS) as control. Hamsters were challenged on 0 dpi (days post-infection; referred to as primary infection) and re-challenged (referred to as secondary infection) with a heterologous SARS-CoV-2 virus (either Ancestral, Alpha, Beta, or Gamma) on 21 dpi. Animals were euthanized by CO_2_ on days 2, 5, and 7 post primary infection. Selected tissues were collected at necropsy for histology and downstream analysis. All infectious work were conducted under approved containment level-3 (CL-3) conditions at the NRC's CL-3 facility.

The following SARS-CoV-2 isolates were used in this study: hCOV-19/Canada/ON-VIDO-01/2020 (B, Ancestral) (National Microbiology Laboratory, Winnipeg, Canada), hCoV-19/England/204820464/2020 (B.1.1.7, Alpha, NR-54000), hCoV-19/USA/MD-HP01542/2021 (B.1.351, Beta, NR-55282), hCOV-19/Japan/TY7-503/2021 (P.1, Gamma, NR-54982), hCoV-19/South Africa/CERI-KRISP-K040013/2022 (Lineage BA.5; Omicron Variant), hCoV-19/USA/MD-HP40900/2022 (Lineage XBB.1.5; Omicron Variant) were obtained through BEI Resources, NIAID, NIH. Viruses were propagated in Vero E6 cells and quantified in Vero cells. Sanger sequencing of the spike gene was carried out to confirm exact genetic identity to original isolate. Passage 2 or 3 virus stocks were used in all subsequent experiment.

### Plaque assay

Virus burden was quantified by plaque assay within the NRC's CL-3 biocontainment facility. Whole left lung was homogenized in 1 ml of 1X phosphate buffered saline (PBS). The plaque assay, in brief, was carried out by diluting the clarified lung homogenate supernatant in a 1:10 serial dilution in infection media (1X DMEM, high glucose media supplemented with 1X non-essential amino acid, 100 U/mL penicillin–streptomycin, 1 mM sodium pyruvate, and 0.1% bovine serum albumin). Vero cells were infected for 1 h at 37 °C before the inoculum was removed and overlay media was added, which consisted of 1X infection media with 0.6% ultrapure, low-melting point agarose). The assay was incubated at 37 °C/5% CO_2_ for 72 h. After incubation, cells were fixed with 10% formaldehyde and stained with crystal violet. Plaques were enumerated and PFU was determined per gram of lung tissue.

### Plaque reduction neutralization test (PRNT)

The PRNT assay was performed in the NRC’s CL-3 facility. Serum samples were inactivated at 56 °C for 30 min and stored on ice. The inactivated serum was serially diluted 1:2 and incubated with equal volume of 100 PFU of SARS-CoV-2 at 37 °C for 1 h, followed by infection of Vero cells. Adsorption of virus were carried out for 1 h at 37 °C. After adsorption, inoculum was removed and cells were overlaid with media as described above. The assay was incubated at 37 °C/5% CO_2_ for 72 h. Cells were fixed with 10% formaldehyde after incubation and stained with crystal violet. No serum, virus-only back-titer control was included along with naïve animal serum. PRNT50 is defined as the highest dilution of serum that results in 50% reduction of plaque-forming units. The 1:2 dilution of diluted serum to 100 PFU virus was included in the final calculation.

### Microneutralization assay

The microneutralization assay was performed in the NRC’s CL-3 facility. Hamster serum samples were inactivated at 56 °C for 30 min and stored on ice. In brief, the inactivated serum was serially diluted 1:5 and incubated with equal volume of 125 PFU of SARS-CoV-2 at 37 °C for 1 h. After incubation, Vero-E6 cells seeded in 96 well plates were infected with sera/virus mixture and adsorption of virus was carried out for 1 h at 37 °C. After adsorption, inoculum was removed and cells were overlaid with media containing serum dilution. The assay was incubated at 37 °C/5% CO_2_ for 72 h. The plates were examined under a brightfield microscope for cytopathic effect (CPE). The microneutralization titer is determined as the highest dilution factor with no detectable CPE.

### Cytokine profiling in hamsters using quantitative real-time PCR

Lung total mRNA was extracted using RNA miniprep kit (Cat # R1058, Zymo Research, Irvine, USA) according to the manufacture’s instruction. 500 ng of RNA was used to synthesize cDNA using reverse transcriptase Super Script III (Cat # 1808-044, Thermofisher, Ottawa, Canada). Cytokine profiling by qRT-PCR was performed in duplicate using SYBR Master mix (Cat # 434446, Applied BioSystems, MA, USA). Fold change gene expression was calculated using ΔΔCt against PBS (mock) infected hamsters as baseline with 18S rRNA as the housekeeping gene. Five animals were analyzed for each experiment. The primer sequences were designed as previously described [56]

### Quantitative real-time PCR of viral genomic RNA

Viral RNA from hamster lung tissues were extracted using Quick-viral RNA kit (Cat #R1035, Zymo Research, Irvine, USA). The extraction was done according to the manufacture’s instruction. Viral RNA was quantified by Luna Universal one-step RT-qPCR kit (Cat #, E3005S, New England Biolabs, MA, USA) with primer/probe sets specific designed for the SARS-CoV-2 E gene (F: ACAGGTACGTTAATAGTTAATAGCGT, R: ATATTGCAGCAGTACGCACACA, Probe: ACACTAGCCATCCTTACTGCGCTTCG [5′]Fam [3′]BHQ-1) Ct values were compared to the SARS-CoV-2 stock (Ancestral) which allowed us to quantify the levels of RNA. Finally, the results were presented as RNA copy numbers/g Lung tissue.

### RBD-specific IgG ELISA

Nunc MaxiSorp flat- bottom 96 well plates were coated with recombinant SARS-CoV-2 RBD- His recombinant protein (40595-V80H, Sino Biological, China) and incubated overnight at 4 °C.

After the incubation, plates were washed with PBS containing 0.1% Tween-20 plates were blocked with 3% Bovine Serum Albumin (IgG-Free). Hamster serum was diluted (fivefold serial dilution) from 1:100 up to 1:1562500. Diluted serums were added to the plate and incubated for 1 h at 37 °C. Next, plates were washed with PBS-T and Peroxidase AffiniPure Goat Anti-Syrian Hamster IgG (H + L) (Cat # 107-035-142, Jackson Immuno Research, West Grove, USA) was added to each well and incubated at 37 °C for 1 h. After the last wash with PBS-T, 100 µL of Tetramethylbenzidine (TMB) substrate (Cat# 7004P6, Cell Signaling Technology, MA, USA) was added to each well. After a two-minute incubation at room temperature, 100 µL of Stop solution (Cat# 7002P6, Cell Signaling Technology, MA, USA) was added to terminate the reaction and absorbance was measured at 450 nm. Inhibitory dilution 50 (ID50) was calculated using non-linear regression analysis.

### Histopathology and immunohistochemistry

All four lobes of the right lung of infected hamsters were isolated and immersed in 10% neutral buffered formalin. After fixation for 1 week at room temperature, lungs were transferred into 70% ethanol, processed and embedded in paraffin wax. The paraffin blocks were cut into 5 µm sections and placed on Superfrost Plus slides (Fischer Scientific). Sections were dried overnight and duplicate sections were subjected to hematoxylin and Eosin (H&E) or immunohistochemistry (IHC) staining.

For H&E staining a fully automated Leica ST5010-CV5030 system was used. Whole slide H&E images were scanned at 20× magnification on a Zeiss Axio Scan.Z1 digital slide scanner capable of brightfield imaging. Histopathology scoring was performed using the criteria described by lien et al. [28]and the samples were blindly scored by a certified pathologist. Briefly, at scanning magnification the percentage of lung area affected by inflammation was estimated. Subsequently the distribution of lesions, extent of the inflammation as well as type of cell infiltrate was scored as described in Table 1.

**Table 1:** Lung histopathology scoring criteria

Clinical score	Histological findings
0	No significant morphological changes
1	Minor inflammatory changes with sparse mononuclear cell infiltration, mainly peribronchial/bronchiolar and perivascular
2	More apparent interstitial mononuclear inflammatory infiltration, alveolus septa thickening, focal areas of consolidation
3	Increased infiltration of inflammatory cells, multiple focal consolidation, diffuse alveolar damage
4	Extensive collapse of alveolar spaces, alveolar septa thickening, more inflammatory cell infiltration in alveoli, larger areas of consolidation, diffuse alveolar damage
5	Similar findings to 4, but the lung tissue is almost completely consolidated

For immunohistochemistry, a modified protocol F on the Bond-Max III fully automated staining system (Leica Biosystems, Wetzlar) was employed. All reagents from the Bond Polymer Refine Detection Kit (DC9800) were used. To characterize the immune cell infiltrates in infected lungs, primary antibodies that cross-react with hamster immune cell antigens [43] were: rabbit polyclonal antibodies against IBA1 (ionized calcium binding adaptor protein, 1), MPO (myeloperoxidase, 1:1000, Dako A0398) and CD3 (1:500, Dako A0452). SARS-CoV-2 was detected using mouse anti-SARS-CoV-2 nucleocapsid monoclonal antibody (1:5000, R&D System MAB10474). Following deparaffinization and rehydration, sections were pre-treated with the Epitope Retrieval Solution 1 (ER1, Citrate buffer, pH 5.0) or Epitope Retrieval Solution 2 (ER2, EDTA buffer, pH 8.8) at 98 °C for 20 min. Epitopes were exposed using ER1 for MPO, IBA1 and SARS-CoV-2 whereas ER2 was used for CD3. After washes, non-specific endogenous peroxidases were quenched using peroxidase block for 5 min. Sections were washed again and then incubated for 15 min at room temperature with primary antibodies. A mouse-on-mouse superblock was applied for 15 min prior to addition of anti-SARS-CoV-2 nucleocaspid antibody (PowerVision IHC/ISH Super Blocking, Leica Biosystems PV6122). After addition of primary and subsequent washes, sections were incubated with polymer refine for 8 min at room temperature and developed with 3, 3′-diaminobenzidine (DAB) chromogen for 10 min. Sections were then washed and counterstained for 6 min with hematoxylin, dehydrated, cleared and mounted. Negative controls included omission of primary antibody and incubation with secondary antibody alone as well as lung tissue from naïve animals.

#### Image analysis

IHC slides were scanned at 20× magnification using a Zeiss Axio Scan.Z1 digital slide scanner capable of brightfield imaging. QuPath 0.3.2, an open-source software for bioimage analysis https://qupath.github.io; [3] was used to detect and count immune-positive cells in whole section brightfield images. Briefly, images were dragged and dropped into the created project folder. Image type was then set to brightfield (H-DAB), which identifies individual cells based on the sum of the hematoxylin and DAB channels. The entire section was carefully selected using the wand tool and immunopositive (stained with hematoxylin and DAB) and negative cells (stained with hematoxylin only) were assigned at this step. The number of immunopositive cells were then calculated using the following steps: Analyze_cell detection_positive cell detection_run. Annotations, as well as markup images of detected cells for visual validation, were then exported into an excel file and the data therein was used for graphing results. It is important to note that the parameters for optimal cell detection and analysis such as threshold value etc. were determined beforehand for each antibody stain, i.e., SARS-CoV-2 nucleocapsid protein, MPO, IBA1 and CD3, and those values were kept constant for all sections. The number of positive cells and area detected were used to calculate the average number of positive cells per mm2.

### Statistics

Data were analyzed using GraphPad Prism® version 9 (GraphPad Software). Statistical significance of the difference between groups was calculated by one-way (ANOVA) followed by post-hoc analysis using Tukey’s (comparison across all groups) multiple comparison test. Data was log transformed (except for % neutralization and % body weight loss) prior to statistical analysis. For all analyses, differences were considered to be nonsignificant with *p* > 0.05. Significance was indicated in the graphs as follows: *p < 0.05, **p < 0.01, ***p < 0.001 and ****p < 0.0001.

## Results

### Alpha variant was most pathogenic and showed lethality in infected hamsters

**Fig. 1:**
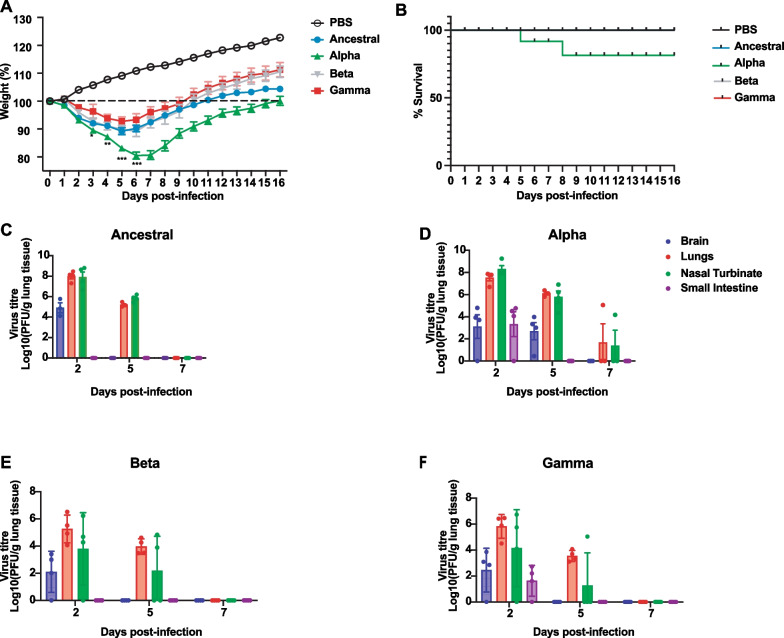
Alpha infection induces higher levels of pathogenicity compared to Ancestral infection in hamsters. Golden Syrian hamsters were intranasally challenged with different SARS-CoV-2 variants; 8.5 × 10^4^ PFU of virus was used for the challenge. Animal's weight was monitored on a daily basis, necropsy was performed on days 2, 5, and 7 post-infection to determine infectious virus titer in different tissues. **A** Weight percentage of hamsters challenged with PBS (Mock), Ancestral, Alpha, Beta, and Gamma. **B** Survival rate of challenged hamsters. **C**–**F** Infectious viral titer in multiple organs including brain, lung, nasal turbinate, and small intestine was measured by plaque assay. **C** Ancestral. **D** Alpha. **E** Beta. **F** Gamma. * Indicates *p* value < 0.05; ** indicates *p* values < 0.01; *** indicates *p* values < 0.001

We examined the pathogenicity of SARS-CoV-2 variants Alpha, Beta, and Gamma in male Golden Syrian hamster as compared to Ancestral strain. Animals were infected intranasally with 8.5 × 10^4^ PFU and weight change was followed over a period of 16 days post-infection (dpi). Control animals were mock infected with PBS. Infected animals showed weight loss as early as 2 dpi for all virus strains with peak weight loss at 5 and 6 dpi. At 6 dpi, males infected with Alpha strain showed the greatest weight loss of average 19.5% compared to only 9.78%, 11% and 8% of original body weight for Ancestral, Beta and Gamma infected animals, respectively (Fig. 1A). Moreover, mortality in hamsters was only observed in Alpha-infected animals (Fig. 1B). Hamsters infected with Ancestral, Beta, and Gamma variants all survived up to the experimental endpoint of 16 days post-infection; however, Alpha infected hamsters showed a survival rate of 77% (Fig. 1B).

We determined the viral burden in various tissues of these infected animals on days 2, 5, and 7 post infection. Respiratory tissues, including lung and nasal turbinate, showed highest virus burden for all VOCs tested. Alpha or Ancestral infected hamsters (Fig. 1C–F) showed highest titer at ~ 10^8^ PFU/g, whereas Beta and Gamma was determined to have ~ 10^6^ PFU/g of tissue at 2 dpi and gradual reduction in viral burden is observed up to 7 dpi. All animals cleared infectious virus by 7 dpi with the exception of Alpha-infected animals, where a small fraction of animals failed to do so. One in 5 Alpha-infected animals failed to clear the infection from the respiratory tissues (Fig. 1D). Lethality is often seen in these animals. This was not observed in any of the other VOCs.

Importantly, the VOCs demonstrate different viral burden and duration. Lower levels of infectious virus, ~ 10^3^ and 10^2^ PFU/g, was detected in the small intestines of Alpha- and Gamma-infected animals, respectively, at 2 dpi, (Fig. 1D, F) but this was not observed in Ancestral and Beta infections (Fig. 1C, E). Of significance was the detection of live, infectious virus from the brains of all VOC-infected animals on day 2 post-infection but not on day 5 or day 7, with the exception of Alpha-infected animals, where detection was still observed up to 5 dpi but completely cleared by 7 dpi (Fig. 1D). Overall, these results demonstrate that, not only was the Alpha variant the most pathogenic variant to emerge during the early days of the pandemic, but also different pathogenicity and tissue burden was exhibited by each VOCs.

### IgG response from different SARS-CoV-2 infections cross-neutralize heterologous variants of concern

**Fig. 2:**
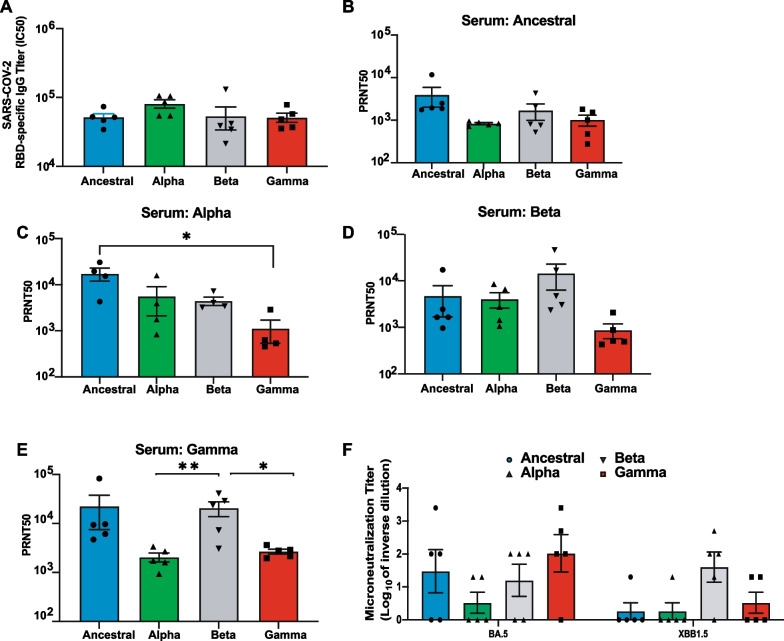
Memory B cells generated after SARS-CoV-2 infection neutralize heterologous SARS-CoV-2 variants of concern. **A** RBD-specific IgG titer was determined using serums from infected hamsters 21 dpi. **B**–**E** PRNT was conducted using hamster serums collected on 21 dpi against SARS-CoV-2 variants. The 50% plaque reduction neutralization test (PRNT50) is defined as the highest serum dilution resulting in 50% reduction in plaque formation units. **B** PRNT50 of serum samples collected from animals infected with Ancestral. **C** PRNT50 of serum samples collected from animals infected with Alpha variant. **D** PRNT50 of serum samples collected from animals infected with Beta variant. **E** PRNT50 of serum samples collected from animals infected with Gamma variant. **F** Microneutralization assay was performed using hamster serums collected on 21 dpi against Omicron variants BA.5 and XBB.1.5. The neutralization titer is determined as the inverse of the highest dilution that does not show CPE

Hamsters infected with different VOCs showed similar measure of RBD-specific, neutralizing IgG level in serum collected at 21 dpi (Fig. 2A). However, the question remained if cross-neutralizing activity could be observed for different VOCs in the serum of recovered hamsters. Cross-protection to heterologous SARS-CoV 2 variants was demonstrated by plaque reduction neutralization test (PRNT) with 21 dpi hamster serum (Fig. 2B–E). There are reports that convalescent serum of individuals infected or vaccinated with the Ancestral strain have reduced neutralization activity to VOCs [48]. While we do see a slight decrease in PRNT50 titer with VOCs from Ancestral-infected convalescent serum (Fig. 2B), the difference was not significant, possibly due to the low number of animal samples tested in this study. Interestingly, serum from Alpha-infected hamsters showed significantly low neutralizing activity to Gamma when compared to its ability to neutralize Ancestral (Fig. 2C). On the other hand, we observed similar neutralizing titer between Ancestral, Alpha and Beta. Unlike Alpha, Beta-infected serum was capable of neutralizing all tested virus strains (Fig. 2D). While Gamma showed a lower neutralizing titer compared to Beta, the difference was not statistically significant (Fig. 2D). Interestingly, serum from Gamma-infected hamsters demonstrated strong neutralization to Ancestral and Beta variants compared to Gamma and Alpha, but similar neutralization levels was observed between Gamma and Alpha (Fig. 2E). Since the identification of these early VOCs, 2 more VOCs have emerged with the Omicron as the latest dominant strain causing current global infections [47]. We, therefore, determined whether infections from these early VOCs still elicit cross-protection against the more recent Omicron sublineages BA.5 and XBB.1.5 (Fig. 2F). We saw a trend in lower neutralizing activity for all sera to BA.5 and XBB1.5. Ancestral-infected animal sera had 3 of 5 animals showing some neutralizing activity to BA.5 compared to only 1 of 5 animals for XBB1.5. Similarly, 4 of 5 Gamma-convalescent sera showed ~ 2.5-log neutralizing titer to BA.5 compared to only 2 animals having titer of about 1.3-log for XBB1.5 (Fig. 2F). Beta-convalescent sera appeared to retain similar levels of neutralizing activity to BA.5 and XBB1.5 (Fig. 2F). Of note, that many of these observations did not achieve statistical significance due to the small sample size of the study. Nevertheless, our data demonstrated that induction of RBD-specific IgG titer was similar for all tested VOCs and Ancestral virus, unrelated to the pathogenicity of the virus. While we did not observe significant reduction in neutralization activity in most of the convalescent sera of animals infected with the Ancestral or the VOCs, possibly due to the low number of animal samples used in the study, we did see a trend that suggested neutralizing activity varies between variants. Importantly, our observations provided evidence that cross-neutralization was retained for all heterologous strains.

### SARS-CoV-2 variants showed different immune profile with Alpha variant inducing stronger inflammatory response in infected hamsters

**Fig. 3:**
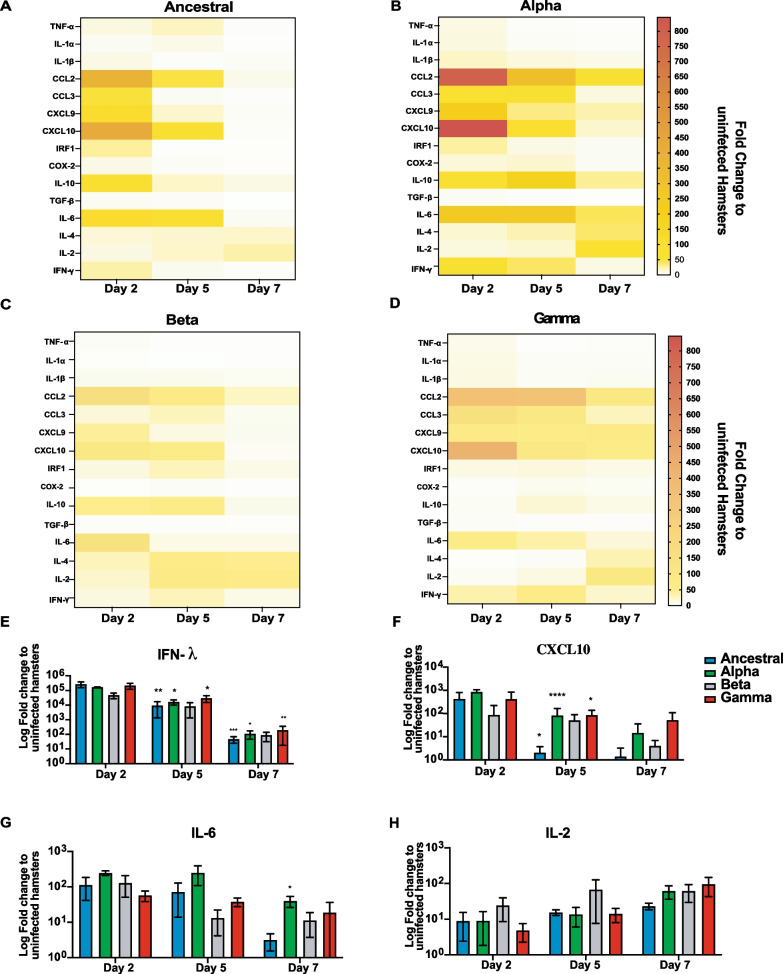
Gene expression analysis of the lung tissue in hamsters infected with different SARS-CoV-2 variants correlated with viral clearance by 7 dpi. **A **Ancestral,** B **Alpha,** C **Beta,** D **Gamma heatmaps of qRT-PCR using RNA extracted from lung tissues from infected hamsters on 2, 5, and 7 dpi. Specific primers against 15 cytokine were designed and used. Fold-change of gene expression was calculated using ΔΔCt against control hamsters administered with PBS. Gene expression was normalized to18S rRNA housekeeping gene. **E** Expression of IFN-λ from 2, 5, and 7 dpi is indicated as fold-change to uninfected control hamsters administered with PBS. **F** Gene expression levels of CXCL10. **G** gene expression levels of IL-6. **H** gene expression levels of IL-2. *p* value on top of each bar indicates the statistical significance compared to 2 dpi of the same group of animals infected with the same variant. * Indicates *p* value < 0.05; ** indicates *p* values < 0.01; *** indicates *p* values < 0.001

Previous studies show SARS-CoV-2 infection triggers a strong cytokine response in COVID-19 patients that lead to pulmonary lung pathology [12]. To better understand the differences in the immune response induced by different SARS-CoV-2 variants, we performed lung mRNA cytokine profiling on days 2, 5, and 7 post infection (Fig. 3). We investigated differential gene expression of pro-inflammatory cytokines (IL-1α, IL-1β, CCL2, CCL3, CXCL9, CXCL10, IRF-1, TNF-α) and anti-inflammatory cytokines (COX2, IL-10, IL-4, IL-6, and TGF-β, Type II IFN: IFN-γ; and type III IFN response: IFN- λ) in infected lung tissues (Fig. 3A–D). Overall immune response for all animals infected with different VOCs and Ancestral virus showed gradual reduction in level of expression in the majority of cytokines examined from 2 to 7 dpi (Fig. 3A–D). Pro-inflammatory cytokine expression peaked at 2 dpi and declined by 7 dpi. IFN-λ expression showed relatively similar, steady elevated levels as early as 2 dpi to 5 dpi, and declined on average ~ 75-fold at 7 dpi (Fig. 3E) for all tested viruses. Infection with all variants induced elevated pro-inflammatory chemokine expression such as CCL2 and CXCL10 at 2 dpi but elevated expression was sustained longer in Alpha- and Gamma-infected tissues as observed at 5 dpi compared to Ancestral (Fig. 3A–D, F). CXCL10 expression showed greatest rate of decline in Ancestral virus infected hamsters compared to the VOCs, with nearly a 2-log drop from 2 to 5 dpi (Fig. 3F), and remained relatively higher in Gamma-infected animals at 7 dpi. IL-6 expression also declined between 2 to 7 dpi though not as much and as quickly as the other cytokines with only sixfold reduction in expression levels in Alpha-infected animals (Fig. 3G), with levels remaining relatively high in Alpha-infected animals even at 7 dpi (50-fold increase). The opposite trend was true for IL-2 and IL-4 with detected increased expression as infection progressed from 2 to 7 dpi (Fig. 3H). Ancestral infection showed an eightfold increase in IL-2 expression at 2 dpi to 24-fold at 7 dpi (Fig. 3A), whereas Alpha infection resulted in a higher level of expression with ninefold increase at 2 dpi to 61-fold increase in expression at 7 dpi (Fig. 3B). Overall, cytokine gene expressions showed a downward trend from 2 to 7 dpi. Alpha and Gamma showed longer elevated expression up to 5 and 7 dpi compared to Ancestral for CXCL10 and CCL2 chemokines.

### Alpha and Beta VOC cause acute lung injury later in disease progression compared to Ancestral and Gamma

**Fig. 4:**
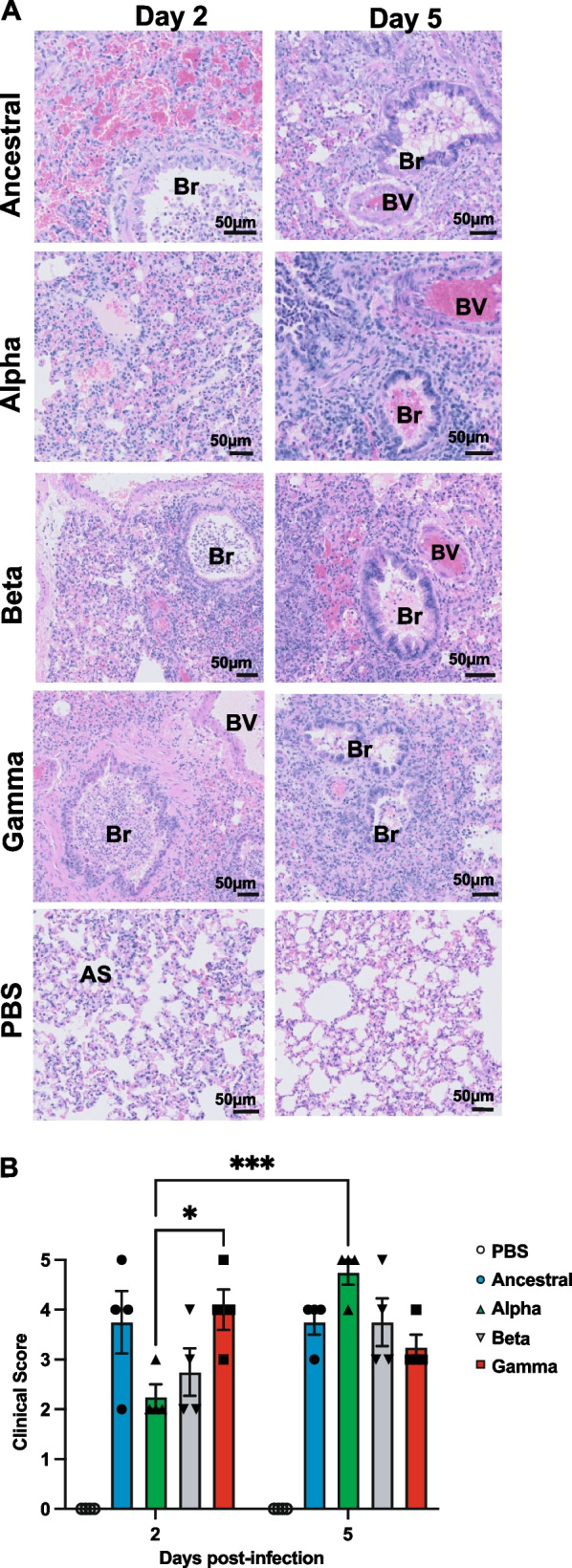
Histopathology of hamster lungs infected with the ancestral and SARS-CoV-2 variants of concern. **A** Representative photomicrographs of hematoxylin and eosin (H&E) stained lung sections at 2 and 5 days post-infection. Infected lungs showed differences in the severity of interstitial pneumonitis, hemorrhage, consolidation of lung parenchyma, and alveolar septal thickening (AS). Increased leukocyte infiltration was seen in bronchioles (Br) and blood vessels (BV). Control hamsters inoculated with PBS showed normal alveolar architecture. **B** Summary of histopathological scores. Lung lesions, extent of the inflammation and mononuclear infiltrate was scored as described in Table 1 on H&E stained samples. Data shown is mean ± SEM from 4 hamsters per group. * Indicates *p* value < 0.05; ** indicates *p* values < 0.01; *** indicates *p* values < 0.001

Histopathological changes were assessed on days 2 and 5 post-infection by examining H&E stained lung sections of infected hamsters. Ancestral virus caused severe acute lung injury as early as 2 dpi with severe multifocal lung consolidation, thickening of alveolar septa, and exudates within the bronchioles and blood vessels (Fig. 4A). Prominent inflammatory infiltrate was apparent throughout the lung parenchyma and surrounding the blood vessels and bronchioles. Infiltrates consisted largely of neutrophils and macrophages, and fewer lymphocytes (Fig. 4A). Similar severe lung injury was also observed in Gamma infected animals. In contrast, lungs from Alpha and Beta infected hamsters showed mild to moderate pneumonia at this early time point (2 dpi), with low mononuclear cells infiltrate around airways and blood vessels, mild thickening of alveolar septa, and the occasional presence of a mixture of neutrophils and macrophages within the airway lumen. Nevertheless, by 5 dpi, all infected animals showed similar lung injury severity (Fig. 4B). As anticipated, mock infection with PBS showed normal, healthy lung morphology. Overall, SARS-CoV-2 variants caused varying degrees of lung pathology at different stages of infection, with the most severe damage observed in Alpha infected lungs (Fig. 4B).

**Fig. 5:**
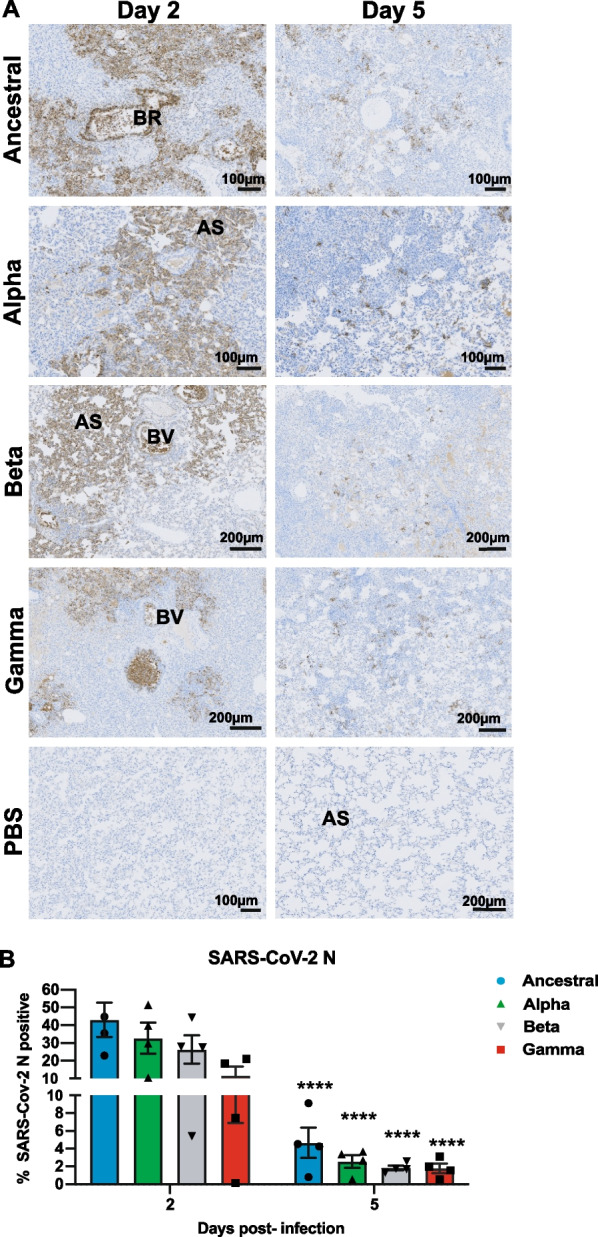
Immunohistochemical detection of SARS-CoV-2 nucleocapsid antigen in hamster lungs infected with Ancestral and variants of concern. Infected hamster lung tissues were stained using anti-SARS-CoV-2 nucleocapsid protein antibody. **A** Representative photomicrographs of nucleocapsid protein expression (dark brown) at 2 and 5 dpi are shown. Nucleocapsid protein was localized mainly to interstitial macrophages in the consolidated areas, alveolar spaces (AS) and bronchiolar epithelium. The lumen of bronchioles (Br) and blood vessels (BV) also show extensive immunoreactivity in the inflammatory infiltrate. **B** Quantitative analysis of nucleocapsid protein staining on IHC images from infected lungs at 2 and 5 dpi using Qupath software as described in Materials and Methods section. Data shown is mean ± SEM of 4 hamsters per group. * Indicates *p* value < 0.05; ** indicates *p* values < 0.01; *** indicates *p* values < 0.001

We also determined viral nucleocapsid antigen levels in the infected lung tissues by IHC (Fig. 5A). Most robust detection of nucleocapsid antigen was determined at 2 dpi, which mainly localized to multifocal patches of large consolidated areas of pulmonary interstitium in macrophages, as well as in cellular exudate within the lumen of pulmonary bronchi, alveolar septa and bronchial epithelial cells (Fig. 5A). Lungs infected with Ancestral strain had the highest level of detectable nucleocapsid protein. Approximately 40% of the cells were immune-positive and this number decreased by tenfold to ~ 4% by 5 dpi (Fig. 5B). Regardless of severity of lung pathology, the infected hamsters were able to clear the viral infection substantially by 5 dpi, with very little nucleocapsid antigen remain detectable in the respiratory tissue for all tested virus strains (Fig. 5A, B).

**Fig. 6:**
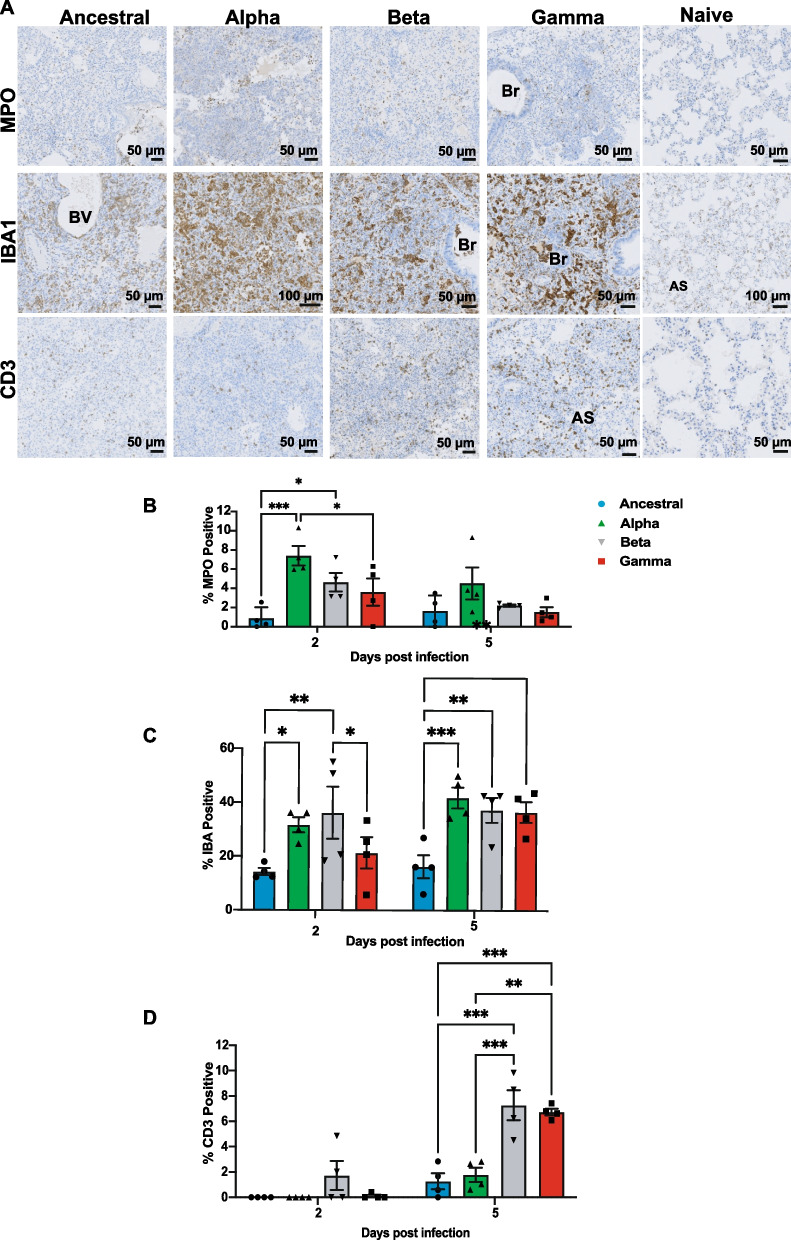
Immunohistochemical detection of inflammatory cell infiltrate in infected hamster lungs infected with Ancestral and variants of concern. Infected hamster lung tissues were stained using anti-MPO (neutrophils), anti-IBA1 (macrophages) and anti-CD3 (T lymphocytes) antibodies. **A** Representative photomicrographs of MPO expression in neutrophils (dark brown) at 5 dpi are shown. **B** Representative photomicrographs of IBA1 expression in macrophages (dark brown) at 5 dpi are shown. **C** Representative photomicrographs of CD3 expression in T lymphocytes (dark brown) at 5 dpi are shown. By 5 dpi, neutrophils, macrophages and T lymphocytes were mainly found in consolidated areas. **D** Quantitative analysis of MPO, IBA1 and CD3 staining on IHC images from infected lungs at 2 and 5 dpi using Qupath software as described in Materials and Methods section. Data shown is mean ± SEM of 4 hamsters per group. * Indicates *p* value < 0.05; ** indicates *p* values < 0.01; *** indicates *p* values < 0.001

A growing number of studies have reported an overt inflammatory response following SARS-CoV-2 infection which is characterized by an increased transmigration of inflammatory cells into the lung parenchyma [12]. Lung tissues from infected hamsters were probed with anti-MPO (major component of azurophilic granules of neutrophils), which has been used as a marker of neutrophil infiltration in tissues [24], anti-IBA1 (ionized calcium binding adaptor protein 1) that specifically binds to macrophages, and anti-CD3 (pan T-cell marker), which is expressed in all T lymphocytes. Similar to previous reports, we also observed elevated neutrophils, expressing high levels of MPO, infiltration throughout the lung parenchyma and also in cellular exudates within the vasculature and lumen of pulmonary bronchi (Fig. 6A). All three VOCs induced higher neutrophil infiltration compared to Ancestral virus, with the greatest observed number of neutrophil infiltrate detected in Alpha-infected animals at 2 dpi (Fig. 6B). Likewise, a massive increase in the number of IBA1-positive macrophages was seen in consolidated areas, localized specifically to perivascular areas and pulmonary interstitium (Fig. 6A, C). Unlike neutrophils, which saw a slight decline by 5 dpi (Fig. 6B), tissue infiltration of CD3 + T-lymphocytes was not observed at elevated levels until 5 dpi (Fig. 6A, D). T lymphocytes were mainly distributed within the pulmonary interstitium and adventitia around bronchioles. We also noted the variants induced immune infiltration at varying levels. For example, Alpha-infected animals showed highest neutrophil infiltration among all three variants, while Beta and Gamma infection resulted in elevated levels of CD3 + T-lymphocytes that was not observed in Alpha-infected animals at 5 dpi (Fig. 6B, D). Altogether, our results demonstrate that the variants of concern engaged stronger innate and adaptive immune responses as compared to the ancestral strain.

### Infection-induced immunity protects hamsters from secondary heterologous infection with no indications of antibody-dependent enhancement of disease

Multiple waves of emerging SARS-CoV-2 variants resulted in individuals becoming infected and re-infected by different VOCs. This raised the question whether immunity to subsequent infections by heterologous strains is affected by the first infection, a phenomenon known as the original antigenic sin [1, 32, 58]. We wanted to determine if initial infection with one variant affects immune protection to subsequent infection with a different variant.

**Fig. 7:**
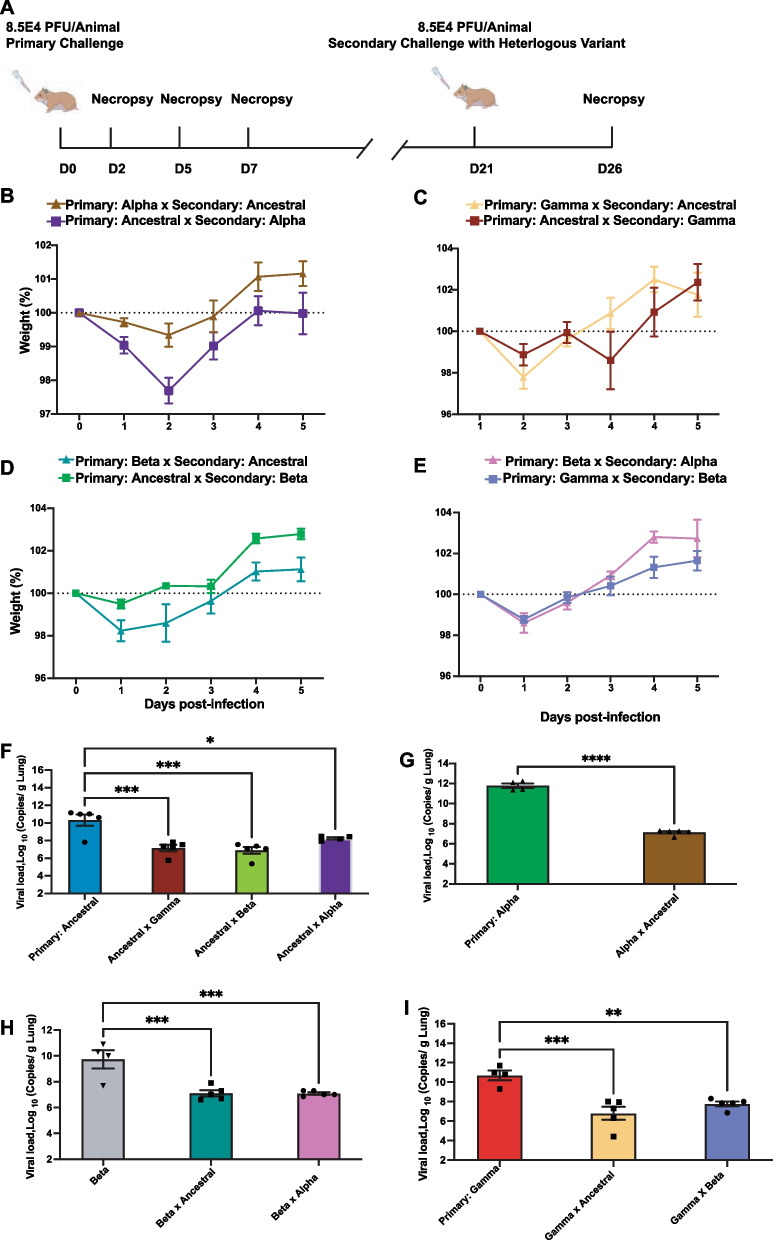
Primary infection induces cross-protective immunity in hamsters rechallenged with heterologous SARS-CoV-2 variants. At 21 dpi, hamsters were rechallenged intranasally with heterologous VOC (8.5 × 10^4^ PFU). **A** Schematic figure of the cross challenge study with heterologous variants in hamsters. **B**–**E** Weights were monitored on a daily basis to 5 dpi with heterologous variant. *p* value is calculated using ordinary two-way ANOVA to calculate the mean difference between different heterologous rechallenged combination. **F–I** RT-PCR was performed on hamster lung samples to quantify SARS-CoV-2 viral loads at 5 days post-primary infection in comparison to 5 days post-secondary infection with heterologous SARS-CoV-2 variants. *p* value is calculated using ordinary one-way ANOVA. *p* values are indicated above the symbols. * Indicates *p* value < 0.05; ** indicates *p* values < 0.01; *** indicates *p* values < 0.001

Hamsters were rechallenged 21 days after primary infection intranasally with a heterologous SARS-CoV-2 variant and monitored daily up to 5 days after the second infection (Fig. 7A). We observed that initial infection with a SARS-CoV-2 variant, regardless of the specific variant, prevent disease severity of the hamsters from a subsequent infection with a different variant, with recovery observed as early as 2 days-post rechallenge (Fig. 7). However, animals that were first infected with the Ancestral virus showed slightly more weight loss when rechallenged with Alpha (2.31% of their weight at 2 dpi), than if the animals were first infected with Alpha and rechallenged with Ancestral (< 1% of their weight, Fig. 7B). The opposite was true for Beta, where we saw greater weight loss of animals initially infected with Beta followed by a rechallenge with Ancestral (Fig. 7D). On the other hand, no difference was observed for animals initially infected with Gamma (Fig. 7C). Similarly, primary infection with Beta or Gamma, followed by a rechallenge with Alpha or Beta, respectively, equally protected the animals (Fig. 7E). This infection-induced protective immunity also translated to significant reduction in viral burden in the respiratory tissues for all infection combinations tested (Fig. 7F–I). Overall, this demonstrates that naturally-acquired immunity from initial infections with any of the tested SARS-CoV-2 variants induced protective immunity to subsequent re-infection with a heterologous variant; that subsequent re-infection did not demonstrate ADE. However, initial infections by certain variants demonstrate slightly better protection compared to others.

**Fig. 8:**
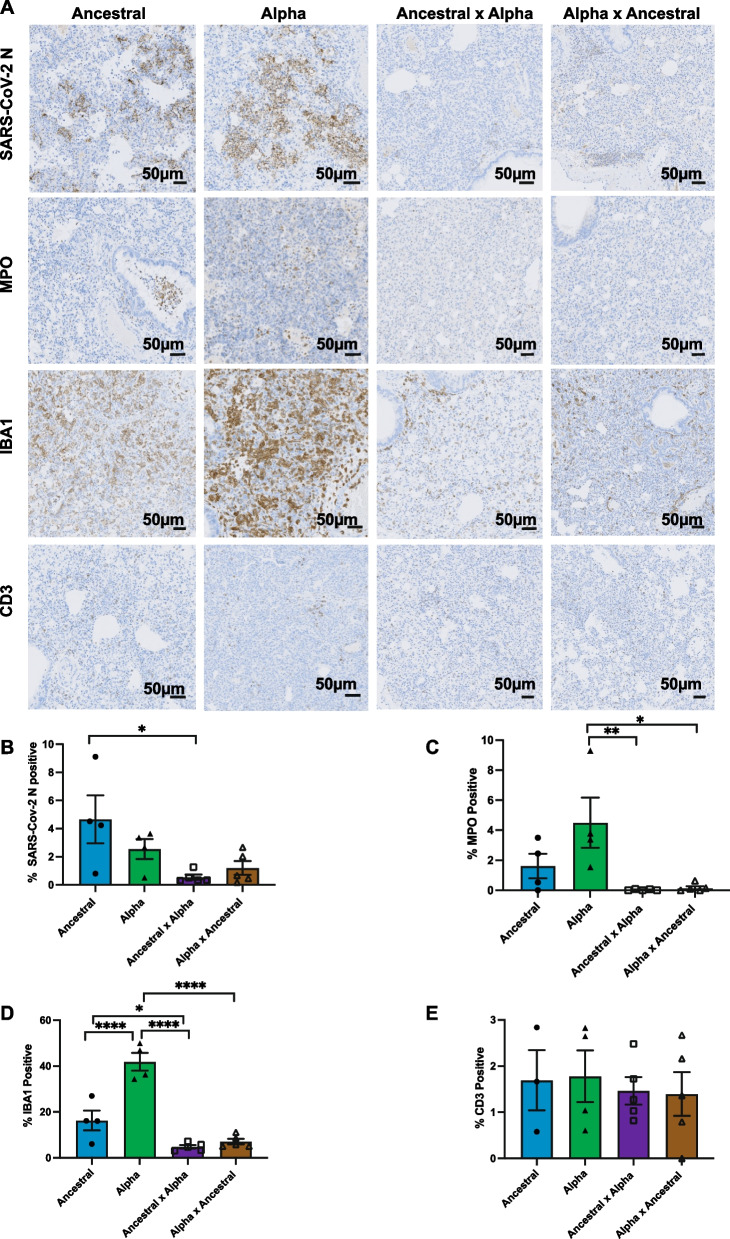
Lung pathology followed by secondary SARS-CoV-2 infection with Alpha variant. Infected hamster lung tissues were stained using anti-MPO (neutrophils), anti-IBA1 (macrophages) and anti-CD3 (T lymphocytes) antibodies. **A** Representative photomicrographs of MPO expression in neutrophils (dark brown) at 5 dpi are shown. **B**–**E** Quantitative analysis of MPO, IBA1 and CD3 staining on IHC images from infected lungs at 2 and 5 dpi using Qupath software as described in Materials and Methods section. Data shown is mean ± SEM from 4 hamsters per group. * Indicates *p* value < 0.05; ** indicates *p* values < 0.01; *** indicates *p* values < 0.001

We next wanted to determine whether the observed protection from a second re-infection also minimize the immune reactivity observed in the initial infection. IHC staining was used to detect cells that are positive for SARS-CoV-2 nucleocapsid (N), IBA1 (neutrophil), MPO (macrophage), and CD3 (T-cell) markers (Fig. 8). Nucleocapsid staining in lung tissue at 5 days post-reinfection corroborates our observation that naturally-acquired immunity reduced viral burden in the respiratory tissues and offered protection of the host (Fig. 8A, B). For example, lung tissues of animals rechallenged with Alpha showed less than 1% with detectable viral antigen, compared to 4.6% of SARS-CoV-2 positive cells in naïve animals infected with Ancestral or 2.5% with Alpha (Fig. 8B). Most notable was the significant reduction of infiltrating inflammatory immune cells, neutrophils and macrophages, into the lung milieu upon re-infection in animals with prior acquired immunity (Fig. 8A, C, D). Interestingly, previous infection did not affect T cell response and level of infiltration into the lungs (Fig. 8E). And immune response was not affected by which variants the host was first infected. Overall, prior infection induced protective immunity that minimized aberrant virus-induced inflammatory response and immune infiltration in the respiratory tissue but maintained similar cell-mediated response.

## Discussion

COVID-19 pandemic saw the emergence of multiple waves of different SARS-CoV-2 variants with accumulating mutations that proffered the virus with varying levels of fitness. As such, individuals have become infected multiple times with different variants but very few studies have been carried out to understand the effect of multiple heterologous infections within a host. While the WHO had declared the pandemic as officially over, the continued infection and emergence of new variants remain a risk to global health. In this study we characterized the different pathogenicity and immune profile of three different VOCs in Golden Syrian hamsters. We further investigated the effect of multiple infections within a single host, and whether the initial infection affects the immune response to subsequent infection with a heterologous variant. Our data demonstrated that Alpha variant was the most pathogenic variant compared to Ancestral, Beta, and Gamma. Infection with Alpha variant resulted in significant weight loss with some hamsters showing lethality (a survival rate of 77%), delayed viral clearance, and pronounced cytokine gene expressions. Thus far, only Alpha variant infections have shown lethality in hamsters compared to the three other SARS-CoV-2 viruses we have tested (Ancestral, Beta, and Gamma). Our findings are in agreement with the literature and support the evidence that the Alpha variant might be the most virulent variant to have emerged thus far as demonstrated in hamsters and mice [22, 50, 51].

We demonstrated that induction of RBD-specific IgG titer was similar for all tested VOCs and Ancestral virus, unrelated to the pathogenicity of the virus. While we did not observe significant reduction in neutralization activity in most of the convalescent sera of animals infected with the Ancestral or any of the VOCs to each other, possibly due to the low number of animal samples used in the study. However, we do see a trend that supports prior studies suggesting a slight reduction in neutralizing capability of certain convalescent sera to specific isolates tested [14, 33, 59]. Nevertheless, our observation provides evidence that cross-neutralization is retained for the most part for all heterologous strains.

When this study was conducted, Delta and Omicron variants had not emerged; therefore, we were not able to include these more recently emerged VOCs in our in vivo challenges. However, we did evaluate if the convalescent sera from these infected hamsters were able to neutralize the more recent Omicron sublineages BA.5 and XBB1.5. In line with reports that infection or vaccination with previous SARS-CoV-2 strains resulted in reduced neutralizing activity to these recent Omicron subvariants [37, 53], we observed substantial decline in neutralizing activity to BA.5 and especially to XBB1.5. Only 1 out of 5 animals showed any detectable levels of neutralization to XBB1.5 for Ancestral and Alpha strains. Interestingly, sera from Beta-infected animals showed at least 2-log neutralization titer that is unchanged for both BA.5 and XBB1.5 with 3 or 4 animals out of 5 showing detectable levels of neutralization. Of the initial VOCs that emerged, Beta was reported to have the highest resistance to neutralization by Ancestral-elicited sera, suggesting antigenic differences [10, 29]. Moreover, studies have determined that Omicron shared mutations K417N and N501Y with Beta [8]. A study that analysed different antibody subsets elicited by Beta infection revealed that a certain population of those neutralizing antibodies retained the ability to also neutralize Omicron, targeting key mutations that are shared between the two variants [41]. This supports our observation that Beta-elicited sera retains low levels of neutralizing activity to both BA.5 and XBB1.5.

The analysis of cytokine profiling and gene expression in lung tissue of infected hamsters indicate that each variant induced distinct cytokine and chemokine profiles. Infection by Alpha resulted in significant increase of several inflammatory cytokines such as CCL2, CXCL10 and IL-6 over a longer period of time compared to Ancestral strain. Gamma variant also showed sustained elevated CXCL10 and CCL2 chemokines compared to Ancestral infection. Elevated levels of CXCL10 and IL-6 has been shown to correlate with severe COVID-19 [23]. CCL2 has a critical role in monocyte infiltration and furthering lung tissue damage; increased levels of CCL2 by Alpha infection is consistent with tissue damage observed in lung pathology of infected hamsters [39]. High pulmonary expression of type III IFNs is detected in critical COVID-19 cases. IFN-λ is the dominant IFN produced in respiratory tissues against viral infection to suppress the viral spread [6]. We observed equally high levels of IFN-λ expressed in the respiratory tissues infected with all tested variants. The levels of the IFN-λ peaked at day 2 post infection and reduced by day 7 post infection; however, even at day 7 post infection, the levels of IFN-λ remain the highest compared to other IFNs and cytokines.

We profiled immune infiltration in the respiratory tissues by IHC. Due to a lack of reagents to carry out in-depth immune characterization in hamsters, our investigation was limited to a few immune markers for activated macrophages, neutrophils, and T lymphocytes. Our data indicated a stronger neutrophil response upon Alpha infection. Neutrophils are one of the earliest immune cells to be recruited and activated during viral infection. Previous study has shown an increase in the number of circulating neutrophils in lung tissue of COVID-19 patients; this increase in number of neutrophils was correlated to severity of COVID-19 [27]. Therefore, an increased number of neutrophils in lung tissue by Alpha infection may indicate a higher pathogenicity of Alpha variant compared to other variants. T cell response plays a key role in protection against severe COVID-19 disease; moreover, with the emergence of SARS-CoV-2 variants of concern and their capability to escape neutralizing antibodies; T cell immune response is critical in containing the infection [54]. Our IHC data shows that there was a notable increase in CD3^+^ T lymphocytes infiltration in the lung tissue at 5 dpi but little was detected earlier. Importantly, we observed different levels of T lymphocyte infiltration from different variants. Beta and Gamma induced stronger CD3^+^ T lymphocyte response compared to Alpha and Ancestral infection. Prior study has shown that T cell immunity is not disrupted by the mutations in variants [49]; the differences in T cell response detected in our IHC study might reflect the longer time needed for alpha and ancestral infection to produce T cell immunity. Further investigations are needed to study the molecular mechanism underlying these immunological differences triggered by different SARS-CoV-2 variants of concern and to better understand the host-virus interaction. These findings provide insights into immunomodulatory interventions that can be used as therapeutics to treat COVID-19.

Previous studies have shown that primary infection in hamsters protects the animal from re-infection [7, 11, 20]. Our re-infection data is in line with prior observations that reinfection results in lower viral loads and limited viral replication in hamsters. Re-infected hamsters did not lose any noticeable weight compared to primary infection where animals lost up to 18% of their weight by 5 dpi. SARS-CoV-2 viral load was detected in lung tissue of re-infected hamsters; however, the quantified viral load was substantially lower compared to primary infection, which suggests that initial infection with SARS-CoV-2 provides a certain degree of immunity to secondary infection with another SARS-CoV-2 variant. Significantly, we observed that immunity acquired from the initial infection greatly reduced the infiltration of immune cells into the respiratory tissue and also promoted strong CD3^+^ T lymphocyte recruitment to the area of infection, regardless of which variant caused the initial infection.

Our data with Ancestral, Alpha, Beta and Gamma, strongly suggests that naturally-acquired immunity provides protection from subsequent infection with a heterologous SARS-CoV-2 strain. This would minimizes disease severity, viral burden, as well as reduces aberrant immune response that lead to severe pathology. Our study provides evidence that infection with a heterologous strain confers protection with no observable enhancement of disease. Moreover, our observations underscore the need to better understand the pathogenicity and host immune response to each variance in order to predict the nature of new emerging SARS-CoV-2 variants, which would permit more informed decisions about future vaccine design, strategy and intervention.

## Conclusion

We showed here that naturally acquired immunity protected hamsters from subsequent re-infection with heterologous SARS-CoV-2 variants, regardless of which variant the animal was first exposed to, and that cross-immunity did not exacerbate disease in subsequent re-infections. Cross-protection in these hamsters did not only result in a decrease in viral burden in the respiratory tissues but also reduced immunopathology and immune infiltrates, while promoting strong, protective CD3^+^ T lymphocyte recruitment to the area of infection, regardless of which variant caused the initial infection. This study provides evidence that underscore the need to better understand the pathogenicity and host immune response of each variant in order to predict the nature of new emerging SARS-CoV-2 variants. Such information is essential to our understanding of the heterogeneity of clinical outcomes in COVID-19 disease and guide vaccine strategy and public policy to emerging SARS-CoV-2 VOCs and future novel pandemic coronaviruses.

## Data Availability

The data presented in this study are available on request from the corresponding author.

## Abbreviations

CPE: Cytopathic effect
VOC: Variants of concern
MAIT: Mucosal-associated invariant T cells
NRC: National Research Council Canada
PFU: Plaque forming unit
PBS: Phosphate buffered saline
dpi: Days post-infection
CL-3: Containment level-3
IHC: Immunohistochemistry
ADE: Antibody-dependent enhancement
